# The Effect of Childhood Eye Disorders on Social Relationships during School Years and Psychological Functioning as Young Adults

**DOI:** 10.22599/bioj.111

**Published:** 2018-05-10

**Authors:** Carol Y. Buckley, Jason C. Whittle, Lily Verity, Pamela Qualter, Judith M. Burn

**Affiliations:** 1 Orthoptic Department, Royal Preston Hospital, Lancashire Teaching Hospitals NHS Foundation Trust, Sharoe Green Lane, Fulwood, Preston, PR2 9HT, GB; 2 The School of Psychology, University of Central Lancashire, Preston, Lancashire, PR1 2HE, GB

**Keywords:** Occlusion, Glasses, Psychological Impact, Social Relationships, Victimisation, Anxiety, Bullying, Childhood

## Abstract

**Aims:**

To determine (1) whether having a visible eye condition and/or treatment with glasses and/or occlusion in childhood has any impact on psychological and/or social outcomes during childhood and young adulthood and (2) whether there is an effect of age at treatment.

**Methods:**

A cohort of 160 participants was asked to take part in an online study. The cohort had previously taken part in a research study at Royal Preston Hospital from 1999–2006 when they were 3–8 years old (Buckley and Perkins 2010). Participants were divided into treatment and no-defect (control) groups and were invited to take part in the current study when they had reached age 18–21. Thirty-five (35) participants (22.5% of the total cohort) were recruited and completed a series of online questionnaires assessing recalled victimisation at school, current generalised anxiety, current depressive symptoms, current loneliness, current friendship quality, and adjustment to university/work. Questionnaire scores between treated patients and controls were compared.

**Results:**

Findings showed that young adults who received treatment during their pre-school years, compared to their peers who did not need treatment, reported higher current generalised anxiety and more victimisation when in school.

Those who received treatment in reception class were no different on psycho-social functioning compared to their peers; with both groups reporting higher victimisation than average compared to previous studies, and mild rates of anxiety.

**Conclusions:**

It appears that having a visible eye condition or treatment with glasses and/or occlusion commencing at pre-school has long term psychological implications, with scores on victimisation and current anxiety levels being higher for the pre-school treatment group compared to the pre-school no defect (control) group. Treatment plans and advice to parents should consider psycho-social outcomes of proposed treatment.

## Introduction

The social and psychological implications of having an eye condition as an adult have been studied extensively when compared to child and adolescent cohorts (Carlton and Kaltenthaler 2011). In those studies that have examined the latter groups, they report decreased self-esteem (Webber and Wood 2005), depressive symptoms (Pinquart and Pfeiffer 2014), general distress (Choong et al. 2004; Hrisos, Clarke and Wright 2004; Searle et al. 2002; Searle 2000) and conflict within the family home (Holmes 2008). Many of those studies examining child samples tended to include outcome measures reported by parents and carers rather than by the child/adolescent themselves. Using that approach, only limited insight is gained into the experience of wearing glasses and/or having occlusion treatment as a child. Our study attempted to overcome this obstacle by asking the patients themselves (now young adults aged 18–21 years) about their previous social relationships at school and their current psychological functioning to examine how having an eye condition and age of treatment impacts outcome.

When children have been asked directly to comment on how their eye conditions and treatment implications have affected their lives, they often report stigma and negative responses from their peers (Koklanis, Abel and Aroni 2006). This is also acknowledged by Golding et al. (2001) who determined that patch treatment for amblyopia and wearing glasses are both independent risk factors with regards to childhood bullying, as reported by 8 year olds (Golding et al. 2001). Bullying itself is a complex issue and it is often difficult to identify a single cause, but its long-term psychological effects are well documented (Qualter et al.). It is, therefore, important that clinicians have an awareness of any links between the treatment process they are initiating and adverse psychological outcomes in the future. Koklaris and Georgievski (2007) recommended research into this, putting emphasis on patching for amblyopia (Koklanis and Georgievski 2007).

## Methods

This study assessed a cohort of participants who were originally part of a previous study undertaken by Buckley and Perkins (2010) at Royal Preston Hospital (Buckley and Perkins 2010). Buckley and Perkins recruited two cohorts of children: (1) a pre-school group of children aged 3–3½ years born between 1 September 1996 and 31 December 1997, screened at their GP surgery: (2) a group of children in their first year of primary school aged 4–5¼ years born between 1 September 1994 and 31 August 1995, screened in school. In the current study, we contacted those children now that they are adults and collected some psycho-social data.

The School of Psychology at the University of Central Lancashire (UCLan) designed an on-line survey that included published questionnaires and analysed the responses.

*Ethical Implications*. Ethical approval was granted by NHS Health Research Authority – Yorkshire and The Humber-Leeds East Research Ethics Committee. A small number of ethical issues were identified early in the course of the study. The most important of those was the potential for psychological distress experienced by the participant, so support information was given on a debrief sheet at the end of the survey recommending that anyone suffering from distress after completing the study should contact their general practitioner (GP) or ‘Young Minds’ – a child/adolescent mental health service. In line with our ethical approval requirements, we also contacted the participants’ GPs informing them of their patients’ role in the study. Starting the survey implied consent, with participants briefed that they could withdraw from the survey at any time. The ethics committee also asked us to remove the suicidal ideation item from the PHQ-9 and we complied with that request.

*Design*. One-hundred-sixty participants from the previous study (Buckley and Perkins 2010) were invited to take part in this study: 80 patients had received treatment for an eye condition (refractive error, amblyopia, manifest strabismus) or had a noticeable lid condition picked up at school (40) or pre-school (40) screening (At the time of answering the questionnaire they were 20–21 and 18–19 years old respectively). The other 80 participants (40 from school screening and 40 from pre-school screening) were those who underwent screening during the first study, but who did not have an eye defect and had, thus, received no treatment; they formed the control groups. The number of participants for this study was limited by the number of pre-schoolers who had treatment for an eye condition (40), the smallest of the four groups. Although there were more individuals in each of the other groups, only 40 from each were invited to participate so-as-to balance the numbers in each group. In the control groups the participants were selected to match the school attended by those in the treatment groups. Matching the members of the control groups to the treatment groups in terms of school attended provided some control of socio-economic status. In the original screening study (Buckley and Perkins 2010) the GPs surgeries selected served the same areas as the schools chosen so ensuring some balance of socio-economic status.

Inclusion criteria were participation in the previous study, willingness to complete an online survey, ability to use and have access to the internet, and agreement to their GP being informed of their participation in the study.

Additionally no defect should have been found at initial screening (aged 3–3½ years) or at final screening (aged 8 years) in the pre-school control group –and no defect should have been found at initial screening (aged 4¼–5¼ years) or at final screening (aged 8 years) in the school control group. The treatment groups needed to have had treatment with glasses and/or occlusion, and/or have had a noticeable eye condition requiring orthoptic observation.

Exclusion criteria included participants now lacking mental capacity, participants now in prison, potential participants unwilling to complete online survey, having no internet access or inability to use the internet and refusal to consent to GP being informed of their participation in the study.

Each potential participant was allocated a unique identification code, shuffled and anonymised so that only the Orthoptic Department was aware which of the four groups each participant belonged to. This allocation of ID numbers avoided any bias by the Psychology researchers during the scoring and analysing stages of the study. The hospital database was checked for current addresses and GP details, with ethical approval being granted for that. Patients were then contacted via a letter asking for their consent to take part in the study and provided with their ID number, as well as a QR code and html link to gain access to the online questionnaires that formed the survey. Because the survey data were collected online, participants did not need to come into hospital or university to complete the survey. The participants used their unique number to log on to the survey website. On completion of the survey, a notification was sent to the Orthoptic Department and an Amazon voucher worth £10 was dispatched to the patient’s address (Funded by the School of Psychology, UCLan). Due to a low response rate, repeat letters were sent to the same individuals, two months after initial contact, in an effort to increase participant numbers. Addresses of all invitees were re-checked using the hospital database. The initial invitation letters to ten individuals had been returned as “Gone away/Not known” (two from each of the control groups and from the school treatment group and four from the pre-school treatment group) and no updated address could be found, so those ten were not sent a second letter. The second batch of letters was sent immediately prior to university holidays as most of the addresses were still the childhood home addresses. Having contacted all potential participants within the smallest group (preschool treatment), we were unable to recruit replacements without unbalancing the groups.

*Questionnaires completed by participants*. Participants completed a series of eight validated and reliable questionnaires online, with standardized instructions for each being used unless otherwise stated below. The questionnaires were presented in a different order for participants taking the survey. Such random presentation overcomes problems of one questionnaire being influenced by fatigue, but also allowed us to gather information on all measures even where a participant stopped the study before completion of all measures. The College Adaptation Questionnaire (Crombag 1968; Vlaander and van Rooijen 1981) was used to measure the participants’ adaption to university; for participants in work or training on-site, the measure was adapted so that it related to adaptation to that environment. 18 statements, scored on a seven-point scale, measure individuals’ psychological, social and interpersonal adaptation to university or work. Ten of the items reflect poor adjustment (e.g. “I find it hard to get used to life here”); eight items reflect positive adjustment (e.g. “I am glad that I came to study/work here”). The score for the CAQ is the sum of the item scores after reverse coding the ten ‘poor adjustment’ items; high scores on the CAQ represent higher adjustment to university. In the current study, alpha was good at 0.87 suggesting that the measure was a reliable measure among participants in the current study. Generalized anxiety was measured using the GAD-7 (Spitzer 2006) where participants rated seven items on a Likert rating scale, with higher scores representing higher levels of anxiety. Example items on the questionnaire are “Feeling afraid as if something awful might happen” or “Worrying too much about different things”. Previous studies show that the GAD-7 is highly reliable (Delgadillo 2012; García-Campayo 2010). In the current study alpha was good (α = 0.91). The UCLA Loneliness Scale (Russell DW. 1996) was used to measure current levels of loneliness reported by the participants. The UCLA includes 20 statements and a four-point Likert rating scale ranging from 1 (never) to 4 (often). Example items on the questionnaire are “How often do you feel that you have a lot in common with the people around you?” or “How often do you feel that your relationships with others are not meaningful?” The UCLA is highly reliable (Beyers and Goossens 2002; Prinstein, Boergers, and Vernberg 2001) as it was in the current study, α = 0.95. Overt, relational, and reputational victimization and prosocial engagement during school years was measured using the Peer Experiences Questionnaire (Prinstein, Boergers, and Vernberg 2001). Each item asked how often each behaviour had been directed toward the participant during their time at school (e.g. “A teen chased me like he or she was really trying to hurt me”). Participants rated how often each of the 18 statements occurred during their childhood on a five-point Likert rating scale from 1 (never) to 5 (a few times a week); higher scores represented high levels of victimization. No previous studies have included the revised version of the questionnaire, but previous studies using the standard measure reported that PEQ was reliable (La Greca and Harrison 2005; McLaughlin and Hatzenbuehler 2009). The measure showed good reliability across all subscales in the current study (α ≥ 0.87). Participants also completed the Patient Health Questionnaire-8 (Kroenke, Spitzer, and Williams 2001), which includes all the PHQ-9 questions without the final item on self-harm and suicidal ideation. The measure is often used to examine severity of depression in non-clinical populations, and in the current study, data were treated as continuous data, with higher scores being indicative of more depressive symptoms. Example items are “Trouble concentrating on things, such as reading the newspaper or watching television” or “Feeling bad about yourself- or that you are a failure or have let yourself or your family down”. Participants rated each item based on how often that situation had occurred over the past two weeks. The final question “Thoughts that you would be better off dead or hurting yourself in some way?” was not included in line with ethical approval for the study. Previous studies show that the Patient Health Questionnaire has good reliability (Kroenke, Spitzer, and Williams 2001; Cameron 2008). In the current study the reliability was good (α = 0.95). For the GAD-7 and PHQ-8, we did not use cut-off to categorise participants into groups of mild, moderate, and severely depressed/anxious youth; that decision was taken based on the low numbers of participants in each group for statistical analyses.

*Analyses Plan*. The analyses involved three sets of t-test comparisons (1) Pre-school treatment vs. pre-school no defect (control), (2) Reception treatment vs. reception no defect (control), and (3) Pre-school treatment vs. reception treatment), with measures of psychological and social functioning as the dependent variables. Effect sizes (Cohen’s d tests) and their 95% confidence intervals (CI) were also calculated. According to Cohen’s benchmark for effect sizes, a large effect size is deemed 0.8 and above (Cohen J. 1988), although it is advisory, when interpreting effect sizes for small samples, that Cohen’s d confidence intervals are also considered in order to establish the precision of results (Brand and Bradley 2016). Therefore, t-test and effect size results that are accompanied by appropriate confidence intervals will be considered important, and those are ones where zero is not contained within the interval (Nakagawa and Cuthill 2007).

## Results

### Final study sample

While response rate was low, it was similar across the four groups. Initially out of the 160 invited, there were 24 respondents (15%), with a further 12 respondents after the second invitation, who fully completed the survey, making a final response rate of 22.5%. (While 37 participants started the survey, one only completed the first page and withdrew, and another participant was excluded because it was found that, although they were referred following pre-school screening, they did not receive treatment until school age.

Table 1 (below) provides information on the final sample of 35 participants, who completed the full survey. Tables 6 and 7 provide a summary of the treatments received.

**Table 1:** Demographic information of the final study samples and treatment received.

Group	Sex		Education/Work				Treatment							

	Male	Female	College	University	Work	Unemployed	Glasses	Glasses & Occln	Glasses & CI Exercises	Glasses & Squint	Glasses, Occln & Squint	Glasses, squint & Surgery	* Obs

Pre-school treatment group N = 8	3	5	2	4	2	0	3	2	1	0	0	1	*1
Pre-school control group N = 9	3	6	2	4	2	1	0	0	0	0	0	0	0
School treatment group N = 8	6	2	1	4	3	0	5	1	0	0	2	0	0
School control group N = 10	2	8	0	7	3	0	0	0	0	0	0	0	0

*Obs = Observed for a visible lid condition.

Table 2 (below) provides mean information on each of the questionnaires for each study group.

**Table 2:** Means (and standard deviations) for all measures across treatment groups.

Measures	Reception Control N = 10		Reception Treatment N = 8		Pre-school Control N = 9		Pre-school Treatment N = 8	

	Mean	SD	Mean	SD	Mean	SD	Mean	SD

CAQ	89.04	18.26	76.75	21.33	85.00	17.51	79.13	20.24
GAD-7	6.40	6.15	8.13	7.36	3.44	3.97	8.00	4.75
UCLA	41.90	12.37	50.38	12.81	37.75	11.95	43.29	8.71
PEQ Overt Victimisation	1.47	0.85	1.83	0.71	1.11	0.24	1.88	0.87
PEQ Relational Victimisation	2.20	1.15	2.17	1.10	1.78	.97	2.88	1.10
PEQ Reputational Victimisation	2.17	1.41	2.04	0.97	1.33	0.65	2.38	1.00
PEQ Prosocial	3.24	0.94	2.98	0.78	3.33	1.04	2.55	1.04
PHQ-8	6.70	6.53	7.50	6.78	5.56	7.06	6.50	5.78

Examination of differences between treatment and control groups was undertaken using t-tests. T-test results shown in Table 3 revealed lower anxiety scores in the pre-school control group compared to the pre-school treatment group. T-test results also showed differences between preschool treatment and control groups on overt victimisation, reputational victimisation, and relational victimisation, with effect size confidence intervals very close to significance or significant; in each case, the pre-school treatment group scored higher on victimisation compared to their same-aged peers. Table 4 shows there were no psycho-social differences between children receiving treatment in reception compared to their same aged-peers. Comparisons between the two treatments groups (Table 5) shows no significant differences, suggesting that the differences between the preschool treatment group and control group was driven by low scores on anxiety and victimisation of those in the pre-school control group. The average scores for the different victimisation categories in the pre-school control group were not abnormally low and are comparable to average scores from previous studies using the victimisation measure (McLaughlin, Hatzenbuehler and Hilt 2009); it is the case that both treatment groups and the reception control group score higher on the different dimensions of victimisation compared to average scores noted in previous studies. On anxiety, average scores for all groups would qualify as mild anxiety (cut- off at score of five (Delgadillo 2012), although the two treatment groups are both approaching the moderate anxiety cut- off of ten.

**Table 3:** T-tests, Cohen’s d effect sizes, and Cohen’s d 95% Confidence Interval for Preschool Treatment group vs Preschool control group.

Measures	t		p	d		Lower Bound	Upper Bound

CAQ	–0.64		0.531	0.31		–0.71	1.33
GAD-7	2.15	*	0.048	–1.04	^γ^	–2.13	0.05
UCLA	1.01		0.330	–0.53		–1.64	0.58
PEQ Overt Victimisation	2.40	*	0.043	–1.20	**	–2.31	–0.08
PEQ Relational Victimisation	2.19		0.045	–1.06	^γ^	–2.15	0.03
PEQ Reputational Victimisation	2.52	*	0.027	–0.97		–2.05	0.11
PEQ Prosocial	–1.55		0.142	0.75		–0.30	1.81
PHQ8	0.30		0.769	–0.15		–1.16	0.87

*Notes*: *Significant p < .05, **95% Confidence Interval does not include zero, indicating that the effect is statistically significant; **γ** while the 95% confidence interval includes a zero, these results are close to significance. Results not assuming equal variance – For Pre-school treatment versus control Overt Victimisation and Reputational Victimisation, Levene’s test for equality of variance showed F = 25.24, p < 0.001, df for t-test = 15 and F = 5.47, p < 0.034, df for t-rest = 15 respectively. For the UCLA, two participants (one from each group).

**Table 4:** T-test, Cohen’s d effect sizes, and Cohen’s d 95% Confidence Intervals for Reception Treatment group vs Reception Control group.

Measures	t	P	d	Lower Bound	Upper Bound

CAQ	–1.25	0.230	0.59	–0.42	1.60
GAD-7	–54	0.595	–0.26	–1.24	0.74
UCLA	1.42	0.174	–0.67	–1.69	0.34
PEQ Overt Victimisation	0.98	0.344	–0.47	–1.47	
PEQ Relational Victimisation	–0.06	0.951	0.03	–0.96	1.02
PEQ Reputational Victimisation	–0.21	0.834	0.25	–0.88	1.09
PEQ Prosocial	–0.64	0.532	0.26	–0.67	1.30
PHQ-8	0.25	0.803	0.25	–1.11	0.87

**Table 5:** T-tests, Cohen’s d effect sizes, and Cohen’s d 95% Confidence Interval for Preschool Treatment group vs Reception Treatment group.

Measures	t	p	d	Lower Bound	Upper Bound

CAQ	0.23	0.823	0.11	–0.93	1.16
GAD-7	–0.04	0.968	–0.02	–1.07	1.03
UCLA	–1.23	0.239	–0.65	–1.77	0.47
PEQ Overt Victimisation	0.11	0.918	–0.05	–1.00	1.10
PEQ Relational Victimisation	1.29	0.218	–0.65	–0.43	1.72
PEQ Reputational Victimisation	0.68	0.509	0.34	–0.72	1.39
PEQ Prosocial	–0.93	0.370	–0.46	–1.53	0.60
PHQ-8	–0.32	0.756	–0.16	–1.21	0.89

Tables 6 and 7 below offer further information regarding refractive errors, occlusion and visual acuities.

**Table 6:** Treatment Summary: School/Reception Treatment Group.

	First prescription	Age at first prescription	Occlusion Treatment						Visual Acuities at referral (Crowded Cambridge Cards)		Visual Acuities at final check (Linear)		Max angle of squint

			Age at start		Age at end		Home or School		R	L	R	L	

1	R: –1.75/–0.25 L: –1.50/–0.25	6 years & 6/12	n/a		n/a		n/a		6/6	6/9	0.00	0.10	n/a
2	R: +1.0/+0.75 L: +1.0/+1.0	5 years & 4/12	n/a		n/a		n/a		6/9	6/9	–0.10	–0.10	n/a
3	R: +1.25DS L: +1.25DS	5 years & 3/12	?	**	?	**	?	**	3/36	6/9	0.70	–0.14	?35^ Eso
4	R: +2.75DS L: +2.75DS	4 years & 7/12	6 years		7 years		School (+? home)		6/12	6/12	0.70	0.60	Min Eso
5	R: –1.00/–0.75 L: –4.00/–1.00	5 years & 4/12	5 years 5/12		7 years 1/12		Not Stated		6/9	6/60	0.10	0.60	n/a
6	R: –2.50/–0.75 L: –2.50/–0.75	5 years & 6/12	n/a		n/a		n/a		6/24	6/24	–0.12	–0.12	n/a
7	R: –0.75/–0.50 L: +0.50/–1.25	5 years & 3/12	n/a		n/a		n/a		6/9	6/6	–0.12	–0.08	n/a
8	Not available	4 years +	n/a		n/a		n/a		?	?	–0.10	–0.10	n/a

*Notes*: ? ** = Had patching but treatment undergone elsewhere, and details unknown.

**Table 7:** Treatment Summary: Pre-school Treatment Group.

	First prescription	Age at first prescription	Occlusion Treatment			Visual Acuities at referral (Singles)	Visual Acuities at final check (Linear)	Max angle of squint

			Age at start	Age at end	Home or School	R	L	R	L	

1	n/a	n/a	n/a	n/a	n/a	6/6pt	6/6pt	–0.10	–0.10	n/a
2	R: +3.25DS L: +3.00DS	3 years & 6/12	n/a	n/a	n/a	6/6	6/6	–0.10	–0.10	n/a
3	R: +3.25DS L: +4.00/0.50	3 years & 6/12	4 years	4 years & 6/12	Not stated	6/6	6/9pt	–0.10	–0.12	35^
4	R:+2.50/–2.00 L:+2.00/–1.75	3 years & 9/12	n/a	n/a	n/a	6/12+1	6/12+1	–0.10	–0.10	n/a
5	R: +4.50/–1.50 L:+4.50/–1.25	3 years & 9/12	n/a	n/a	n/a	6/12	6/9 +1	–0.12	–0.12	n/a
6^x^	R:+3.25/–0.50 L:+4.25/–0.75	4 years & 9/12	n/a	n/a	n/a	6/6	6/9	–0.10	–0.10	n/a
7	R:+4.50/–1.25 L:+3.00/–0.75	3 years & 5/12	3 years & 10/12	4 years & 10/12	Home (? At school In last 2/12)	6/12 pt	6/18	–0.10	–0.08	n/a
8	R:+1.75DS L:+4.00DS	3 years & 7/12	3 years & 11/12	4 years & 1/12	Home	6/6	6/12+1	–0.12	–0.02	n/a

*Notes*: 6^x^ this individual also had convergence exercises.

## Discussion

The current study set out to examine whether children who had treatment with glasses and or occlusion therapy had poorer social experiences during school and negatively affected psychological functioning, and how the age of treatment impacted those relationships. Findings showed that the young adults in the current sample who received treatment during their pre-school years reported higher current generalised anxiety and more victimisation when they were in school than their same aged peers who did not need treatment for an eye condition. There were no psycho-social differences between children receiving treatment in reception compared to their same aged-peers.

The finding that those wearing glasses and/or patches in pre-school, compared to their same-aged peers, experienced higher levels of victimisation is consistent with previous research, which indicated treatment for refractive errors and/or amblyopia, via the use of glasses and/or patches, to be an independent risk factor for childhood bullying (Koklanis, Abel and Aroni 2006). This outcome was specific to the preschool treatment group, because the difference was the result of low levels of victimisation reported by same-aged peers in the control group; the same differences were not found for the reception class children, where those in the treatment and control groups reported high levels of victimisation comparable to our pre-school treatment group. It is possible that school anti-bullying programmes reduced levels of victimisation reported by children in the pre-school group, but that victimisation was still evident for those children receiving treatment for an eye condition; it is also possible that there are individual differences we have not examined in the study that increased victimisation for the pre-school and reception treatment groups and the reception control group.

It appears that having a visible eye condition and/or treatment with glasses and/or occlusion therapy during pre-school increases the likelihood that children will report victimisation from peers. Our findings show that both victimisation and anxiety are experienced by those who have a visible eye condition or wear glasses and/or occlusion, during pre-school. Presumably some young adults are more susceptible than others. Our research design means we are unable to examine whether later anxiety came from those earlier experiences of victimisation by peers, because of the small sample size in the present study. Given that previous research shows early years’ victimisation (pre-school) is significantly associated with anxiety in later years, we would expect to find evidence for victimisation as a mediator linking having a visible eye-condition/glasses and/or occlusion in childhood to anxiety in late adolescence (Roth, Coles and Heimberg 2002). Fear of negative evaluation is a key component of anxiety and is particularly relevant for the pre-school treatment group who are beginning university (Slee 1994); the reception treatment group have already overcome this life-stage and that is perhaps why they do not report high levels of current anxiety. It should be noted, however, that although non-significant, the reception treatment group had a higher mean score of anxiety than the control group, which may have been significant if we had a larger sample. Thus, future work will want to determine whether the association between eye condition and anxiety as mediated by victimisation is about having treatment rather than the time of that treatment; it may be that having treatment is the leading factor in the occurrence of relational victimisation and, subsequently, anxiety. Future research with larger samples should explore that possibility.

In addition to the eye condition, it may be important to consider the potential victimisation and psychological outcomes that treatment may have.

Studies have already shown that there is no significant ophthalmological benefit/advantage to pre-school treatment as compared to school-age treatment (Buckley and Perkins 2010; Koklanis and Georgievski 2007; Hall and Elliman) and if our findings are supported by further research it would indicate that there may be little benefit psychologically in initiating treatment at pre-school rather than when patients are in school.

Psychological support may be helpful where indicated to help patients cope. All children in both treatment groups (apart from one child in the pre-school treatment group, who had a visible lid condition) wore glasses; therefore psychological effects may be from wearing glasses alone.

Patching done at home may cause a child to be less anxious and distressed; it is more obvious to peers if done in school. None of the participants in our cohort had Atropine occlusion, which was offered much less frequently at that time. It may be that Atropine occlusion, not being as obvious to the peer group, may have less psychological impact than patching therapy. Felius et al. concluded that “Atropine treatment was found to have a less negative impact than patching” (Felius 2010). This finding is important in deciding how to offer and deliver occlusion treatment. It is however, uncertain whether any of the included subjects were actually patched at school.

One factor that was not examined in this study was parenting skills and styles. We feel that this could influence psychological outcomes but investigating that would have the potential to create further ethical issues because such research would involve exploration of the parent-child relationship. Within that examination, researchers may want to examine social support from parents, but also from the wider community, including teachers, other school staff, peers, and friends. Given that having one supportive friend can protect against feelings of marginalisation, having a best friend during childhood may mitigate the negative effects of having an eye condition. It has been shown that “peer support seems to affect well-being of adolescents with a visual impairment” (Kefa and Dekovic 2004).

There are some limitations to the current study. The sample size is small and self-selected, which could mean that those who were more anxious were more inclined to complete the survey. However, the control group of the same age had a comparable response rate, meaning that response bias is unlikely to be a huge issue in the current study. An additional influencing factor may have been the fact that the pre-school group were 18–19 years-old and may have been starting tertiary education or employment, whereas the school group were at a different stage in life. There may be some value in repeating the survey with the pre-school group once they are the age at which the school group were on responding. The small sample size also limited the statistical analyses we were able to conduct, and future work will want to recruit sufficient participants to their studies to ensure adequate power for analyses. In addition to that, understanding exactly which factors (i.e. the eye condition and its treatment) predict victimisation and anxiety may be a useful endeavour, and understanding how school based anti-bullying programmes affect the victimisation experiences of children with eye-conditions compared to their peers will be important. We must also remind the reader that the victimisation measures were modified for the current study to ask participants to think about their experiences when they had been in school. The measure about university adjustment was also adapted so it could be completed by participants in work. Such changes could lead to validation issues in relation to those questionnaires.

## Conclusion

Based on our results – in agreement with previous studies, it appears that having a visible eye condition or treatment with glasses and/or occlusion commencing at both pre-school and school age can have long term psychological implications, more specifically overt victimisation. Additionally, scores on relational victimisation and current anxiety levels were found to be higher for the pre-school treatment group when compared to the pre-school control group. Treatment plans and parental discussion/choice should take potential psychological impact into account.

Suggested areas for future study include increasing the prospective sample size, analysing parental styles and attitudes to treatment, investigating further, using prospective designs, whether Atropine occlusion causes the same negative psychological functioning as patching (Feliu 2010) appears to do in our study, revisiting the pre-school group once they have reached 20–21, researching predictive factors for victimisation; examining the effect of parent-child, teacher-child, and peer-child support.
